# The lost children: The underdiagnosis of dyslexia in Italy. A cross-sectional national study

**DOI:** 10.1371/journal.pone.0210448

**Published:** 2019-01-23

**Authors:** Chiara Barbiero, Marcella Montico, Isabella Lonciari, Lorenzo Monasta, Roberta Penge, Claudio Vio, Patrizio Emanuele Tressoldi, Marco Carrozzi, Anna De Petris, Anna Giulia De Cagno, Flavia Crescenzi, Giovanna Tinarelli, Antonella Leccese, Alessandra Pinton, Carmen Belacchi, Renzo Tucci, Maria Musinu, Maria Letizia Tossali, Anna Maria Antonucci, Anna Perrone, Mara Lentini Graziano, Luca Ronfani, of behalf of the EpiDIt (Epidemiology of Dyslexia in Italy) working group

**Affiliations:** 1 Clinical Epidemiology and Public Health Research Unit, Institute for Maternal and Child Health–IRCCS “Burlo Garofolo”, Trieste, Italy; 2 Child Neurology and Psychiatry Ward, Institute for Maternal and Child Health–IRCCS “Burlo Garofolo”, Trieste, Italy; 3 Child Neuropsychiatry Department, UOC NPI B La Sapienza University, Rome, Italy; 4 Child Neuropsychiatry Unit, Hospital of San Donà di Piave, San Donà di Piave, Italy; 5 Department of General Psychology, University of Padua, Padua, Italy; 6 Department of otolaryngology and Head and Neck Surgery, “Santo Spirito” Hospital, Pescara, Italy; 7 ASL Rm3, Rome, Italy; 8 Centro di neuropsicologia clinica dell'età evolutiva Giorgio Sabbadini, Perugia, Italy; 9 Società Scientifica Logopedisti Italiani (SSLI), Campobasso, Italy; 10 Department of Communication Sciences, Humanities and International Studies, University of Urbino “Carlo Bo”, Urbino, Italy; 11 Studio di Psicologia e Logopedia, Verona, Italy; 12 Associazione Italiana per la Ricerca e l'Intervento nella Psicopatologia dell'Apprendimento (AIRIPA), Padua, Italy; 13 Unità Operativa Complessa NPI ASL Viterbo, Civita Castellana, Italy; 14 Associazione Italiana Dislessia, Bologna, Italy; Hong Kong Polytechnic University, HONG KONG

## Abstract

**Background:**

Developmental dyslexia is one of the most common neurobehavioral disorders affecting children, but prevalence data on this condition are poor. The objective of the present study is to determine the prevalence of dyslexia in Italy in an unselected school population, using clearly defined diagnostic criteria and methods.

**Methods:**

Cross-sectional study carried out in nine Italian Regions: two located in Northern Italy (Friuli Venezia Giulia and Veneto), three in Central Italy (Marche, Lazio and Umbria) and four in Southern Italy (Abruzzo, Molise, Puglia and Sardegna). Three consecutive levels of screening were carried out: the first two at school, to screen the population and identify children with suspect dyslexia; the last in centers with multi-professional staff specialized in learning disabilities to confirm the diagnosis. The key outcome measure is the prevalence of dyslexia, defined as the ratio between the number of children confirmed positive at the third level of screening and the total number of children enrolled in the study.

**Results:**

We finally recruited 11094 children aged 8–10 years, of which 9964 constituted the final working sample after applying exclusion criteria and including only children who received parents’ consent to participate. The prevalence of dyslexia in the whole sample was 3.5% (95% CI 3.2–3.9%), with little differences between Northern, Central and Southern Italy (respectively 3.6%, 3.2% and 3.7%). In almost two out of three children with dyslexia the disorder had not been previously diagnosed.

**Conclusions:**

This study confirms that in primary school children at the age of 8–10 years in Italy dyslexia is widely underestimated. Reliable data on dyslexia prevalence are needed to allocate necessary human and financial resources both to Health Services and Schools, ensuring timely support to children and families.

## Introduction

Developmental dyslexia is among the most common neurobehavioral disorders affecting children. Prevalence data on this condition are poor and often dated. Studies conducted in English-speaking countries show a wide range of prevalence (from 5 to 17.5%), [1–3] mainly due to different methods, tests and definitions adopted for the diagnosis, the type of disability assessed (i.e., dyslexia vs. learning disabilities), and the different ages considered. [4–8] Italy is also affected by this variability with a limited number of studies showing a prevalence ranging from 1.3% to 8.5%. [9–19]

To overcome this situation, in 2008 a group of associations and institutions dealing with dyslexic children in Italy (see Annex) established a National Committee (CENDi) to define methods, instruments and diagnostic criteria to be used in research to accurately ascertain the prevalence of dyslexia. In particular, to confirm the diagnosis, a detailed and unequivocal diagnostic algorithm, combining different tests and cut-offs, was developed and tested in an Italian region (Friuli Venezia Giulia). [20] This study showed that the tools and the methodology defined by the CENDi could be effectively applied to estimate the prevalence of dyslexia. Furthermore, the study showed, in an unselected grade four school population, a large underestimation of dyslexia: prior to the study, out of 1365 children screened, 13 (1%) had a formal diagnosis of dyslexia; at the end of the study, the prevalence of dyslexia rose to 3.1–3.2%. Therefore, dyslexia had not been diagnosed in two out of three children aged 8–10 years. At this age, the disorder is clearly expressed and can thus be identified unequivocally. The non-recognition of two-thirds of the cases of dyslexia has relevant negative consequences at both the clinical and pedagogical levels, translating into insufficient resources for diagnosis, rehabilitation and education.

Given the relevance of these results, for both the importance of early diagnosis and a better allocation of resources to Health Services and Schools, the CENDi decided to extend the study to the national level.

## Material and methods

A cross-sectional study was carried out between 2008 and 2013 in nine Italian Regions: two located in Northern Italy (Friuli Venezia Giulia and Veneto), three in Central Italy (Marche, Lazio and Umbria) and four in Southern Italy (Abruzzo, Molise, Puglia and Sardegna). At the national level, Centers and Associations expressed their interest in participating in the study. Thus the study was conducted in selected areas of their reference Regions. In each Region a coordinator was identified from the participating Centers and Association. Furthermore, local supervision was ensured by the psychologist (Chiara Barbiero) who coordinated the pilot study in Friuli Venezia Giulia. The study methodology has been extensively described elsewhere. [20] Briefly, the sample consisted of children aged 8–10 years attending the 4^th^ year of the Italian primary school. Children were excluded if: 1) certified with mental retardation by local health authorities according to Italian Law n° 104/92 (framework law on disabled persons); [21] 2) not of Italian nationality; 3) had been absent from school for more than two months since 1^st^ grade.

Starting at the beginning of the school year, three consecutive levels of assessment were carried out to reach a diagnosis of dyslexia.

The first level was carried out at school by specifically trained psychologists, and adopted the following tools:

-   A short anamnestic questionnaire to be filled in by parents, with questions concerning the child and his/her family (age, language spoken at home, health status of the child, handicap certification and previous diagnosis of Learning Disability, parental formal education level and working status).
-   A questionnaire derived from the validated questionnaire “*RSR-DSA*. *Questionario per la rilevazione di difficoltà e disturbi dell’apprendimento*”, [22] to detect Learning Disabilities (LD), filled in for each child by the classroom teacher.
-   A 4^th^-grade dictation task, derived from the “BVSCO–Battery for the assessment of writing skills in children from 7 to 13 years old”. [23]

Children who scored positive on the teachers’ questionnaire and/or in the dictation task, and who did not fall under the exclusion criteria, moved to the second level. To ensure all children with reading difficulties were identified, teachers were further asked to indicate 1) children who read more slowly than classmates and 2) children who made more reading errors than classmates. All children falling under one of these two criteria were also selected for second level testing. Furthermore, to avoid discrimination from peers, in classes in which children with reading difficulties had been identified, classmates not falling under the selection criteria were also randomly selected for the evaluation at the 2^nd^ level.

The 2^nd^ level evaluation, aiming at the identification of children with reading difficulties and adequate cognitive ability through individual tests, was conducted at school by the same trained psychologists involved in the 1^st^ level evaluation. For individual testing, the following tools were used:

-   word and non-word reading tasks deriving from the DDE-2 Battery (Battery for the assessment of Developmental Dyslexia and Dysorthographia-2), [24] to assess reading speed and accuracy.
-   Vocabulary and Block Design subtests of the WISC-III (Wechsler Intelligence Scale for Children) [25,26] to appraise the cognitive ability of the child.

Children with adequate subtest scores at the WISC-III and poor reading tasks performance were thus selected for the 3^rd^ level of screening.

The 3^rd^ level evaluation aimed to confirm the diagnosis of dyslexia and was carried out in each area at centers with multi-professional staff specialized in the diagnosis of learning disabilities. All children were assessed as follows:

a)  Detailed questionnaire filled in by parents and discussed with a psychologist during an interview. This tool allowed to collect information on the development of the child (gait, autonomy, speech, etc.), kindergarten and primary school attendance (social and communication skills, learning disabilities, etc.), clinically significant events occurred during childhood (i.e., illnesses, injuries), and information regarding the formal education of close relatives (school performance, learning difficulties, etc.).
b)  A test to evaluate the cognitive performance (Raven's Progressive Matrices PM47 or Wechsler Intelligence Scale for Children– 3^rd^ Ed.). [25–28]
c)  MT battery (*Prove di lettura MT per la scuola elementare-2*) to evaluate text reading speed and accuracy. [29,30]
d)  DDE-2 (Battery for the evaluation of Dyslexia and dysorthography-2) to evaluate word and non-word reading speed and accuracy (tasks 2 and 3), and spelling accuracy (tasks 6 and 7). [24]
e)  Strengths and Difficulties Questionnaire (SDQ) administered to parents to evaluate the mental health status of their child. [31]

The criteria adopted to guide the diagnosis of dyslexia by combining the results of these tests is comprehensively described in our previous paper (see also S1 Fig and S1 Table). [20] The study was approved by the Independent Bioethics Committee of the Institute of Maternal and Child Health, IRCCS “Burlo Garofolo”, Trieste, Italy. Before children’s enrollment, an informed consent form was signed by parents.

### Statistical analysis

The sample size was calculated to obtain an accurate estimate of dyslexia prevalence in the three Italian macroregions (Northern, Central and Southern Italy). The total population of children attending 4^th^ grade in Northern, Central and Southern Italy was estimated to be 250.000, 100.000 and 200.000, respectively. Given these data, and under the hypothesis of a 4% prevalence of dyslexia, ranging from 3 to 5%, with a precision of 5% and a power of 80%, a sample size of 1500 children per area was calculated. We decided to enroll an extra 15% to compensate for possible dropouts. A cluster randomization of schools was carried out only in Friuli Venezia Giulia Region. In the others Regions, schools were mainly selected in the reference territory of the participating Centers and Associations. School participation was voluntary.

Continuous data were presented as means and standard deviations, while categorical data as frequencies and percentages.

Prevalence of dyslexia was defined as the number of children positive to the third level of screening (numerator) divided by the total number of children analyzed at the first level.

To estimate the diagnoses of dyslexia in children lost to the third level of follow up, we adopted a multinomial logistic regression analysis, applying the prediction model developed for the Friuli Venezia Giulia Region. [20] Based on this model, these subjects were classified as with or without dyslexia and included in the calculation of the total prevalence.

To evaluate if the enrolled population was representative of the population of the whole participating Regions and, possibly, of the three Italian Macroregions, a comparison between the before study prevalence of dyslexia in children analyzed at the first level and the prevalence estimated by administrative school data (certification of dyslexia known to the school) was made. Administrative data were provided by the Statistical Office of the Italian Ministry of Education, Universities and Research (MIUR) for the school year 2013/14. [18] Unfortunately, only pooled data with all the five years of the primary school were available. Since it is possible to formalize the diagnosis of dyslexia only from the end of the second year of primary school, and consequently to certificate it to the school, to avoid a possible underestimation, the prevalence of dyslexia was calculated using the number of children attending the last three years of primary school as the denominator.

## Results

**Fig 1:**
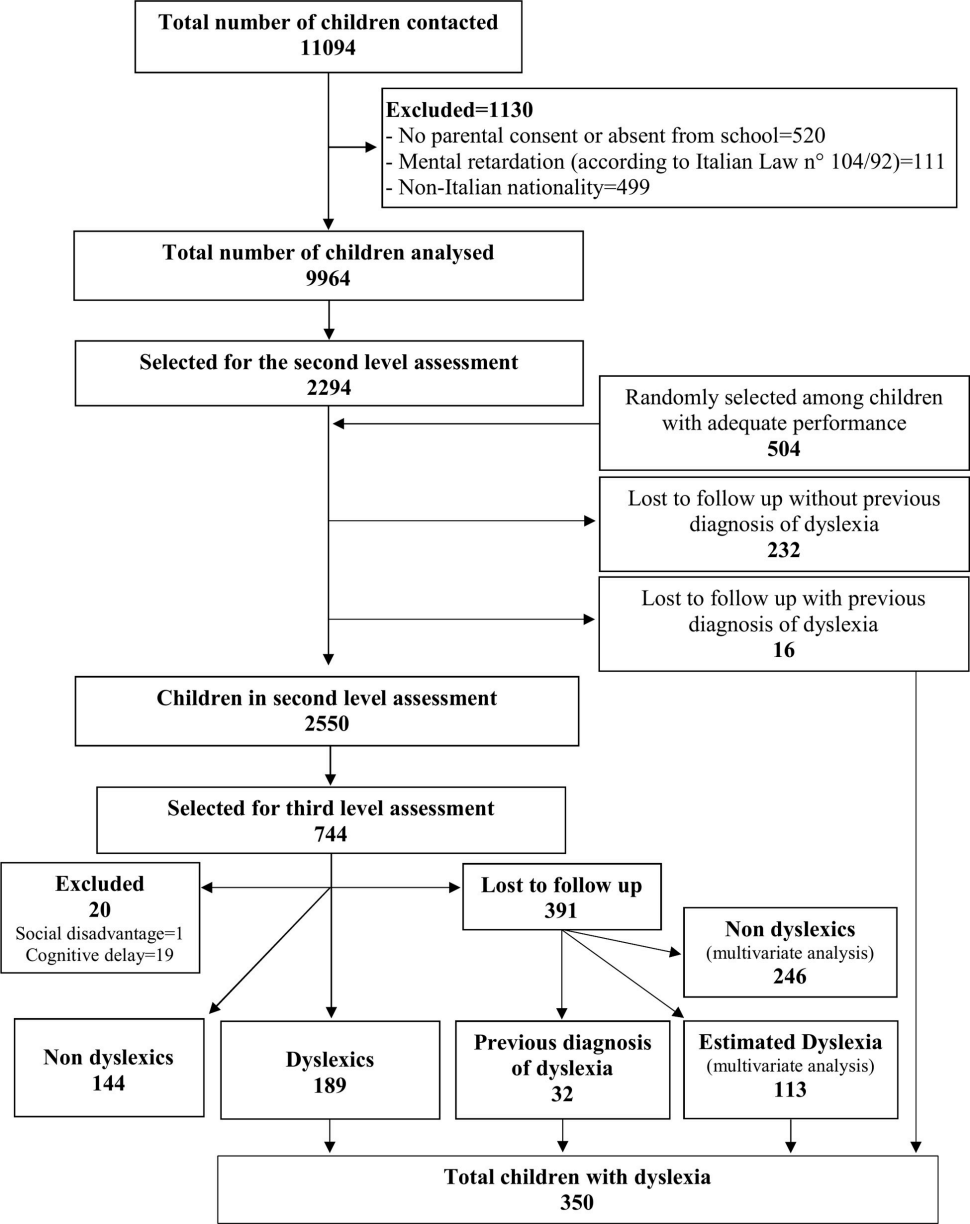
Study flow chart.

A total of 712 grade four classes were enrolled. Overall, 11094 pupils were contacted, and 9964 were analyzed at first level, after exclusion of children without parental consent or absent from school (n = 520), with mental retardation (n = 111) and without Italian nationality (n = 499) (Fig 1).

Two thousand six hundred and forty-nine children were from Northern Italy, 3518 from Central Italy and 3797 from Southern Italy.

### First level evaluation

Characteristics of children evaluated at the first level are presented in Table 1.

**Table 1:** Main characteristics of the selected population.

Variable		Children evaluated (n = 9964)	Data available for (number)
Sex	Female	4679 (49.0%)	9543
	Male	4864 (51.0%)	
Language spoken at home	Italian	8791 (92.0%)	9548
	Dialect	658 (6.9%)	
	Other	99 (1.1%)	
Age of the mother, mean (standard deviation)		40.4 (4.8)	9759
Age of the father, mean (standard deviation)		43.6 (5.6)	9646
Mother’s formal education level	None/elementary	188 (2.0%)	9698
	Lower secondary	2865 (29.5%)	
	Upper secondary	4901 (50.5%)	
	Degree	1744 (18.0%)	
Father’s formal education level	None/elementary	281 (3.0%)	9586
	Lower secondary	3496 (36.4%)	
	Upper secondary	4311 (45.0%)	
	Degree	1498(15.6%)	
Mother with job		6461 (66.7%)	9682
Father with job		9193 (96.0%)	9576
Children with previously formalized dyslexia diagnosis		126 (1.3%)	9964
Children with previously formalized Learning Disabilities diagnosis (dyslexia, dysgraphia, dysorthography, dyscalculia)		282 (2.8%)	9964

The study group is representative of the national population in terms of educational level of the parents (68.5% vs 65.0% Upper Secondary or Degree education in mothers; 61.0% vs 60.0% Upper Secondary or Degree education in fathers) (www.istat.it).

One hundred and twenty-six out of the 9964 children (1.3%) had already received a formal diagnosis of dyslexia.

Two thousand two hundred and ninety-four children scored positive in at least one of the two tests (dictation or teachers’ questionnaire) or were identified by the additional questions addressed to the teachers (n = 215).

### Second level evaluation

Overall, 2798 children were selected for the second level assessment (2294 selected at the first level of screening and 504 randomly selected among children with adequate performance). Unfortunately, 248 children were lost to follow up (of which 16 with a previous diagnosis of dyslexia), and consequently, 2550 children were seen at the second level. Seven hundred and forty-four scored positive in the tests and were selected for the third level of screening.

### Third level evaluation

Three hundred and fifty-three out of 744 children underwent further testing to confirm the diagnosis. Twenty presented cognitive delay (n = 19) and social disadvantage (n = 1) and were consequently excluded; 189 were diagnosed with dyslexia; 144 were classified as not having dyslexia. Among 391 subjects lost to follow up at the third level evaluation, 32 had a previous diagnosis of dyslexia. For the 359 remaining subjects, the logistic model built for the prediction of dyslexia classified as dyslexics further 113 children.

The prevalence of dyslexia was 3.5% (350/9964) (95% CI 3.2–3.9), counting children who fell under the diagnostic criteria (n = 189), children lost to follow up at the 2^nd^ and 3^rd^ level but with a previous diagnosis of dyslexia (n = 48), and children estimated to have dyslexia with the logistic model (n = 113).

The main characteristics of the children with and without dyslexia are reported in S2 Table.

Tables 2 and 3 show the data stratified by the three Italian macroregions before and after the study and the comparison with administrative data provided by the Italian Ministry of University and Research (MIUR). [18]

**Table 2:** Results on prevalence of dyslexia before and after the study and comparison with school administrative data (certification of dyslexia known to the school) for the participating regions.

	Study results Children attending the 4^th^ year of Italian primary schools					MIUR# data School year 2013–2014		
	Children with diagnosis of dyslexia before the study (number)	Children with diagnosis of dyslexia after the study (number)	Population analyzed (number)	Prevalence before the study (95% CI)	Prevalence after the study (95% CI)	Children with certification of dyslexia (number)	Children attending the last three years of the primary school (number*)	Prevalence (95% CI)
Northern Italy Regions (Veneto, FVG)	38	96	2649	1.4% (1.0–2.0%)	3.6% (2.9–4.4%)	2701	170714	1.6% (1.5–1.6%)
Central Italy Regions (Marche, Umbria, Lazio)	58	112	3518	1.6% (1.3–2.1%)	3.2% (2.6–3.8%)	3181	225186	1.4% (1.4–1.5%)
Southern Italy Regions (Abruzzo, Molise, Puglia, Sardegna)	30	142	3797	0.8% (0.5–1.0%)	3.7% (3.2–4.4%)	2300	201469	1.1% (1.1–1.2%)
All Regions	126	350	9964	1.3% (1.1–1.5%)	3.5% (3.2–3.9%)	8182	597370	1.4% (1.3–1.4%)

# Italian Ministry of Education, Universities and Research

* estimate.

**Table 3:** Prevalence of dyslexia in Italy from school administrative data (certification of dyslexia known to the school).

	MIUR# data School year 2013–2014		
	Children with certification of dyslexia (number)	Children attending the last three years of the primary school (number*)	Prevalence of dyslexia (95% CI)
Northern Italy	15199	759177	2.0% (2.0–2.0%)
Central Italy	5085	321787	1.6% (1.5–1.6%)
Southern Italy	4336	615399	0.7% (0.7–0.7%)
Italy	24620	1696363	1.5% (1.4–1.5%)

# Italian Ministry of Education, Universities and Research

*estimate

These data underline that: 1) in the participating Regions the prevalence of dyslexia before the study was similar to that estimated using school administrative data both from the same Regions and from the three Italian macroregions; 2) the prevalence of dyslexia in primary school children is widely underestimated, particularly in the Regions of Southern Italy. Overall, only 1 out of 3 dyslexic children is diagnosed at the age of 8–10 years, and this value rises to almost 5 in Southern Italy Regions (Table 2).

## Discussion

By using rigorous diagnostic criteria and methods, this study extended to the national level the estimate of the prevalence of dyslexia calculated on an unselected school population of a single Italian Region. The results of the pilot study conducted in the Friuli Venezia Giulia Region [20] have been fully confirmed in the new areas involved: dyslexia is not recognized in almost two out of three children at the age of 8–10 years, when the disorder should be clearly expressed and identified. Before the study, 126 out of 9964 children screened had a formal diagnosis of dyslexia, with a prevalence of 1.3%. At the end of the study the prevalence rose to 3.5%. Interestingly, while administrative school data show a north-south gradient in dyslexia prevalence (Tables 2 and 3), with a higher prevalence in Northern Italy, our study shows a similar prevalence in the three Italian macroregions. This means that the level of underestimation of dyslexia is worse in Southern Italy. The consequence of the non-recognition of two-thirds (or more) of the cases of dyslexia is the lack of adequate and timely intervention, leading to internalizing (anxiety and depressive) behaviors, [32–38] suicidal ideation, school failure and drop out. [39,40] Children with specific learning disabilities experience feelings of failure within the school education system, in particular if their problem is not recognized and adequate support is not provided; [41] they can experience poor emotional well being and self-esteem, and a high level of dissatisfaction in their relationships with family and friends. [42] Children with specific learning disabilities are often subject to stigmatization by families, teachers and peers, which can lead to increased self-stigma and reduced motivation to learn. [43,44] Evidence shows that, in children with learning disorders, early diagnosis when associated to appropriately designed interventions, substantially improves self-confidence and social competency, providing better opportunities at school and at work, and consequently improving the quality of life. [36] A timely diagnosis of dyslexia is needed, as also addressed by the Italian law 170/2010, to guarantee the right to education and ensure equal opportunities for capacity building in the social and professional sphere. [45, 46]

As discussed in our previous paper, [20] it is difficult to compare the prevalence obtained in the present study with those previously reported for Italy, given the differences in methods, definitions and diagnostic criteria adopted. [9–17]

Given the lack of adequate *ad hoc* funding, this study was only possible through the voluntary participation of researchers and structures. For this reason, we could not guarantee the complete coverage of the national or regional territory nor carry out a cluster randomization, exposing the study to the risk of selection bias. This is the main study limitation. The comparison with the school administrative data described in Table 2 suggests that the risk of selection bias is very limited for the participating Regions, except for Southern Italy. However, we had expected a positive selection bias, i.e., a higher prevalence of dyslexia in the participating areas given the possible participation in the study of more motivated schools and health professionals. This risk seems not to be present. Table 3 shows that the prevalence of dyslexia at the start of the study in the covered areas is similar to that estimated for the whole macroregions from school administrative data. Another limitation, extensively discussed in our previous paper, is that it was not possible to administer individual reading tests to all children at the 1^st^ level, given the large size of the sample.

The study presents several strengths: the detailed and unequivocal validated diagnostic algorithm, combining different tests and cut-offs, used to confirm the diagnosis; the large sample size; the rigorous application of screening tools by specifically trained staff; the confirmation of the diagnosis performed in centres with multi-professional staff specialized in the diagnosis of learning disabilities to avoid bias in the diagnostic process; the involvement in this process of both child neuropsychiatrists and psychologists.

## Conclusions

This study confirms that in primary school children in Italy dyslexia is widely underestimated: at the age of 8–10 years, in two out of three children, dyslexia had not been previously diagnosed. Reliable data on dyslexia prevalence are necessary to adequately allocate human and financial resources both to Health Services and Schools.

## Supporting information

S1 Fig Procedure for third level screening. (DOCX)

S1 Table Definition of poor performance in reading tasks at the third level evaluation. (DOCX)

S2 Table Main characteristics of the selected population by group (dyslexic vs non-dyslexic children). (DOCX)

S1 Text Contact information for center/association involved in data collection. (DOCX)

## References

[1] Interagency Committee on Learning Disabilities. Learning disabilities: a report to the U.S. Congress Government Printing Office; 1987.

[2] Shaywitz SE, Shaywitz BA, Fletcher JM, Escobar MD. Prevalence of reading disability in boys and girls. Results of the Connecticut Longitudinal Study. JAMA. 1990;264:998–1002. 2376893

[3] Demonet JF, Taylor MJ, Chaix Y. Developmental dyslexia. Lancet. 2004;363:1451–60. 10.1016/S0140-6736(04)16106-0 15121410

[4] Shaywitz SE, Fletcher JM, Shaywitz BA. Issues in the definition and classification of attention deficit disorder. Top Lang Disord. 1994;14:1–25.

[5] Peterson RL, Pennington BF. Developmental dyslexia. Lancet. 2012;379:1997–2007. 10.1016/S0140-6736(12)60198-6 22513218 PMC3465717

[6] Whitaker S. Inner learning disability. Brit J Learn Disabil. 2004;32:139–143.

[7] Katusic SK, Colligan RC, Barbaresi WJ, Schaid DJ, Jacobsen SJ. Incidence of reading disability in a population-based birth cohort, 1976–1982, Rochester, Minn. Mayo Clin Pro. 2001;76:1081–92. 10.4065/76.11.1081 11702896

[8] Berger M, Yule W, Rutter M. Attainment and adjustment in two geographical areas. II-The prevalence of specific reading retardation. Br J Psychiatry. 1975;126:510–9. 1174768 10.1192/bjp.126.6.510

[9] Baldini G, Brasca E.L’apprendimento della lettura e della scrittura: aspetti psicopatologici e considerazioni psicopedagogiche. Infanzia Anormale. 1958;26:167–191.

[10] Faglioni P, Gatti B, Paganoni AM, Robutti A. La valutazione psicometrica della Dislessia. Infanzia Anormale. 1967;81:628–661.

[11] Bisiacchi P, Brotini D, Fornari D. Indagine sull’incidenza della dislessia in un campione di bambini padovani. Formazione e cambiamento. 1978;1:3–16.

[12] Sava D, Buffardini C. Dislessia evolutiva: aspetti socio-ambientali e neuropsicologici. Giornale Italiano di Psicologia. 1981;8:405–419.

[13] Cassini A, Ciampalini L, Lis A. La dislessia in Italia. Strumenti di rilevazione ed incidenza in alcune regioni. Età Evolutiva. 1984;18:66–73.

[14] Levi G, Piredda ML. Strategie semantiche e strategie fonologiche nella costruzione di anagrammi in bambini dislessici. Neuropsichiatria Infantile. 1982;250/251:439–450.

[15] Masala C, Petretto DR, Stella G. Studio epidemiologico sui DSA in una popolazione sarda. Psichiatria dell’Infanzia e dell’Adolescenza. 1998;65:648–653.

[16] Moreno R, Pianta F, Stella G. L’incidenza dei Disturbi Specifici di Lettura nella Scuola Media Superiore: uno studio comparativo. Dislessia. 2005;2:135–146.

[17] Coscarella C. Epidemiologia dei Deficit Specifici di Apprendimento nel territorio dell’Isola d’Elba. Psichiatria dell’Infanzia e dell’Adolescenza. 2001;68:7–15.

[18] Ministero dell’Istruzione, dell’Università e della Ricerca—Ufficio di statistica. L’integrazione scolastica degli alunni con disabilità a.s. 2014/2015. Roma, 2015. Available from: http://www.istruzione.it/allegati/2015/L'integrazione_scolastica_degli_alunni_con_disabilit%C3%A0_as_2014_2015.pdf

[19] Lindgren SD, De Renzi E, Richman LC. Cross-national comparisons of developmental dyslexia in Italy and the United States. Child Dev. 1985;56:1404–17. 3878269

[20] Barbiero C, Lonciari I, Montico M, Monasta L, Penge R, Vio C, et al The submerged dyslexia iceberg: how many school children are not diagnosed? Results from an Italian study. PLoS One. 2012;7(10):e48082 10.1371/journal.pone.0048082 23118930 PMC3485303

[21] Legge-quadro per l’assistenza, l’integrazione sociale e i diritti delle persone handicappate, Public Law n.104 (February 4, 1992). Gazzetta Ufficiale della Repubblica Italiana 1992; 39 (Suppl.Ordinario n.30).

[22] Cappa C, Albanesi E, Muzio C, Guglielmino P, Ferraris V, Barbiero C, et al Uno strumento per la rilevazione di indicatori di rischio di DSA: il questionario RSR-DSA. Dislessia. 2012;9:1.

[23] Tressoldi PE, Cornoldi C. Batteria per la valutazione della scrittura e della competenza ortografica nella scuola dell’obbligo (BVSCO, Battery for the assessment of writing skills of children from 7 to 13 years old). Firenze: Giunti OS; 2000.

[24] Sartori G, Job R, Tressoldi PE. DDE-2. Batteria per la valutazione della dislessia e della disortografia evolutiva (Battery for the assessment of developmental dyslexia and dysorthographia). Firenze: Giunti OS; 2007.

[25] Wechsler D. WISC-III Wechsler Intelligence Scale for Children–third ed. Firenze: Giunti OS; 2006.

[26] Orsini A, Picone L. WISC-III Contributo alla taratura italiana. Firenze: Giunti OS; 2006.

[27] Raven JC. CPM Coloured Progressive Matrices. Firenze: Giunti OS; 2006.

[28] Belacchi C, Scalisi MT Cannoni E, Cornoldi C. CPM Coloured Progressive Matrices. Firenze: Giunti OS; 2008.

[29] Cornoldi C, Colpo G. Prove di lettura MT per la scuola elementare– 2. Firenze: Giunti OS; 2004.

[30] Cornoldi C, Tressoldi PE, Perini N. Valutare la rapidità e la correttezza della lettura di brani. Nuove norme e alcune chiarificazioni per l'uso delle prove MT. Dislessia. 2010;7:89–100.

[31] Goodman R, Ford T, Simmons H, Gatward R, Meltzer H. Using the Strengths and Difficulties Questionnaire (SDQ) to screen for child psychiatric disorders in a community sample. Int Rev Psychiatry. 2003;15:166–72. 10.1080/0954026021000046128 12745328

[32] Mugnaini D, Lassi S, La Malfa G, Albertini G. Internalizing correlates of dyslexia. World J Pediatr. 2009;5:255–64. 10.1007/s12519-009-0049-7 19911139

[33] Willcutt EG, Pennington BF. Psychiatric comorbidity in children and adolescents with reading disability. J Child Psychol Psychiatry. 2000;41:1039–48. 11099120

[34] Nelson J, Harwood H. Learning disabilities and anxiety: a metaanalysis. J Learn Disabil. 2011;44:3–17. 10.1177/0022219409359939 20375288

[35] Törő KT, Miklósi M, Horanyi E, Kovács GP, Balázs J. Reading Disability Spectrum: Early and Late Recognition, Subthreshold, and Full Comorbidity. J Learn Disabil. 2018;51:158–167. 10.1177/0022219417704169 28406742

[36] Törő KT, Balázs J. [The importance of early diagnosis and intervention in children diagnosed with reading disorder. Case studies] [Article in Hungarian] Neuropsychopharmacol Hung. 2015;17:99–103. 26192903

[37] Rutter M. Emotional disorder and educational underachievement. Arch Dis Child. 1974;49:249‑56. 4133779 10.1136/adc.49.4.249 PMC1648762

[38] McGee R, William S, Share DL, Anderson J, Silva P. The relationship between specific reading retardation, general reading backwardness and behavioural problems in a large sample of Dunedin boys: A longitudinal study from 5‑11 years. J Child Psychol Psychiatry. 1986;27:597‑610. 3771677 10.1111/j.1469-7610.1986.tb00185.x

[39] Daniel SS, Walsh AK, Goldston DB, Arnold EM, Reboussin BA, Wood FB. Suicidality, school dropout, and reading problems among adolescents. J Learn Disabil. 2006;39:507–14. 10.1177/00222194060390060301 17165618

[40] Wilson A, Armstrong C, Furrie A, Walcot E. The mental health of Canadians with self-reported learning disabilities. J Learn Disabil. 2009;42:24–40. 10.1177/0022219408326216 19103798

[41] Gibson S, Kendall L. Stories from school: Dyslexia and learners’ voices on factors impacting on achievement. Support for Learning 2010;25:187–193.

[42] Ginieri-Coccossis M, Rotsika V, Skevington S, Papaevangelou S, Malliori M, Tomaras V, et al Quality of life in newly diagnosed children with specific learning disabilities (SpLD) and differences from typically developing children: a study of child and parent reports. Child Care Health Dev. 2013;39:581–91. 10.1111/j.1365-2214.2012.01369.x 22372869

[43] Shifter D. Stigma of a label educational expectations for high school students labeled with learning disabilities. Journal of Health and Social Behavior 2013;54:462–480. 10.1177/0022146513503346 24311756

[44] Chan Y, Chan YY, Cheng SL, Chow MY, Tsang YW, Lee C, et al Investigating quality of life and self-stigma in Hong Kong children with specific learning disabilities. Res Dev Disabil. 2017;68:131–139. 10.1016/j.ridd.2017.07.014 28763755

[45] Nuove norme in materia di disturbi specifici di apprendimento in ambito scolastico, Public Law N.170 (October 8, 2010). Gazzetta Ufficiale della Repubblica Italiana 2010; 244.

[46] Feola A, Marino V, Masullo A, Trabucco Aurilio M, Marsella LT. The protection of individuals affected with Specific Learning Disorders in the Italian Legislation. Clin Ter. 2015;166:e177–181. 10.7417/CT.2015.1851 26152629

