# Supplementary material for: The lost children: The underdiagnosis of dyslexia in Italy. A cross-sectional national study
Source: PLoS One. 2019 Jan 23;14(1):e0210448. doi: 10.1371/journal.pone.0210448 (PMC6343900; doi:10.1371/journal.pone.0210448)
Supplement: S1 Fig — (DOCX) [file pone.0210448.s001.docx]

**Figure S1. Procedure for third level screening**

Assess children selected for third level as follows:

1. Detailed standardized anamnestic questionnaire;
2. Raven’s Progressive Matrices PM47 or WISC III
3. MT battery;
4. Battery for the evaluation of Dyslexia and dysorthography-2 (DDE-2);
5. Strengths and Difficulties Questionnaire (SDQ) for parents.

Apply the following criteria for the diagnosis of dyslexia:

1. Poor performance on reading tasks in at least three out of six scores (see Table S1);
2. Poor performance on reading tasks in at least two scores out of six, in two different reading tests (see Table S1); AND parental acknowledgement of the disorder;
3. Poor performance on reading tests in at least three out of six scores (see Table S1); AND parental recognition of the disorder; AND teachers’ acknowledgement of the disorder;
4. Additional criterion: poor performance in at least one score on non word reading tasks (see Table S1); AND parental recognition of the disorder; AND c) teachers' recognition of the disorder

Exclude children with following features:

1. Cognitive delay;
2. Diseases or sensory and neurological abnormalities;
3. Severe psychopathology;
4. Conditions of environmental, social or cultural disadvantage;
5. Poor teaching.

**Dyslexia diagnosis**

(NB: re-evaluate subjects with uncertain diagnosis)
