# Supplementary material for: The lost children: The underdiagnosis of dyslexia in Italy. A cross-sectional national study
Source: PLoS One. 2019 Jan 23;14(1):e0210448. doi: 10.1371/journal.pone.0210448 (PMC6343900; doi:10.1371/journal.pone.0210448)
Supplement: S1 Table — (DOCX) [file pone.0210448.s002.docx]

**Table S1. Definition of poor performance in reading tasks at third level evaluation**

| **First and second criteria** | |
| --- | --- |
| - DDE-2* | z-score ≤-1.8 (speed) |
|  | OR |
|  | percentile <5° (accuracy) |
| - MT§ | z-score ≤-2 (speed) |
|  | OR |
|  | percentile ≤5° (accuracy) |
| **Third criterion** | |
| - DDE-2* | z-score between -1.8 and -1.5 (speed) |
|  | OR |
|  | percentile between 5° and 10° (accuracy) |
| - MT§ | z-score between -2 and -1.5 (speed) |
|  | OR |
|  | between 5° and 10° |
| **Fourth criterion** | |
| - DDE-2 non word* | z-score ≤-1.8 (speed) |
|  | OR |
|  | percentile ≤5° (accuracy) |

* DDE-2: Battery for the evaluation of Developmental Dyslexia and Dysorthography-2

§ MT: MT battery (Prove di lettura MT per la scuola elementare-2)
