# Supplementary material for: The lost children: The underdiagnosis of dyslexia in Italy. A cross-sectional national study
Source: PLoS One. 2019 Jan 23;14(1):e0210448. doi: 10.1371/journal.pone.0210448 (PMC6343900; doi:10.1371/journal.pone.0210448)
Supplement: S2 Table — (DOCX) [file pone.0210448.s003.docx]

**Table S2. Main characteristics of the selected population by group (dyslexic vs non-dyslexic children)***

| Variable | | Children with dyslexia (n=350)§ | Children without dyslexia (n=9614) | p |
| --- | --- | --- | --- | --- |
| Sex | Female | 136 (41.5%) | 4543 (49.3%) | 0.005 |
|  | Male | 192 (58.5%) | 4672 (50.7%) |  |
| Language spoken at home | Italian | 307 (93.0%) | 8484 (92.0%) | 0.68 |
|  | Dialect | 19 (5.8%) | 939 (6.9%) |  |
|  | Other | 4 (1.2%) | 95 (1.0%) |  |
| Age of the mother, mean (standard deviation) |  | 39.5 (5.0) | 40.5 (4.8) | <0.001 |
| Age of the father, mean (standard deviation) |  | 43.0 (6.3) | 43.6 (5.6) | 0.04 |
| Mother’s formal education level | None/elementary | 24 (7.1%) | 164 (1.8%) | <0.001 |
|  | Lower secondary | 140 (41.7%) | 2725 (29.1%) |  |
|  | Upper secondary | 137 (40.8%) | 4764 (50.9%) |  |
|  | Degree | 35 (10.4%) | 1709 (18.3%) |  |
| Father’s formal education level | None/elementary | 27 (8.2%) | 254 (2.7%) | <0.001 |
|  | Lower secondary | 158 (48.2%) | 3338 (36.1%) |  |
|  | Upper secondary | 122 (37.2%) | 4189 (45.2%) |  |
|  | Degree | 21 (6.4%) | 1477 (16.0%) |  |
| Mother with job |  | 190 (56.5%) | 6271 (67.1%) | <0.001 |
| Father with job |  | 304 (93.0%) | 8889 (96.1%) | 0.005 |

* between groups differences evaluated with the chi-square test for categorical variables and with ANOVA for continuous variables

§ all children with dyslexia, including estimated
