# Supplementary material for: The lost children: The underdiagnosis of dyslexia in Italy. A cross-sectional national study
Source: PLoS One. 2019 Jan 23;14(1):e0210448. doi: 10.1371/journal.pone.0210448 (PMC6343900; doi:10.1371/journal.pone.0210448)
Supplement: S1 Text — (DOCX) [file pone.0210448.s004.docx]

**Contact information for Center/Association involved in data collection:**

Friuli Venezia Giulia Region: Luca Ronfani [luca.ronfani@burlo.trieste.it](mailto:luca.ronfani@burlo.trieste.it)

Veneto Region: Renzo Tucci [renzotucci@yahoo.it](mailto:renzotucci@yahoo.it)

Marche Region: Carmen Belacchi [carmen.belacchi@uniurb.it](mailto:carmen.belacchi@uniurb.it)

Lazio Region: Maria Letizia Tossali [marialetizia.tossali@asl.vt.it](mailto:marialetizia.tossali@asl.vt.it) (Viterbo); Flavia Crescenzi [flaviacrescenzi@inwind.it](mailto:flaviacrescenzi@inwind.it) (Roma)

Umbria Region: Giovanna Tinarelli [g.tinarelli@cnee.it](mailto:g.tinarelli@cnee.it)

Abruzzo Region: Anna De Petris [annadepetris@hotmail.com](mailto:annadepetris@hotmail.com)

Molise Region: Chiara Barbiero [chiara.barbiero@gmail.com](mailto:chiara.barbiero@gmail.com)

Puglia Region: Anna Perrone [coordinamento.puglia@aiditalia.org](mailto:coordinamento.puglia@aiditalia.org)

Sardegna Region: Maria Musinu [mariamusinu@yahoo.it](mailto:mariamusinu@yahoo.it)
